# A systematic evaluation of physical activity and diet policies in Scotland: results from the 2021 Active Healthy Kids Report Card

**DOI:** 10.1093/pubmed/fdae022

**Published:** 2024-02-22

**Authors:** Simone A Tomaz, John J Reilly, Avril Johnstone, Adrienne Hughes, Jenni Robertson, Leone C A Craig, Farid Bardid

**Affiliations:** Faculty of Health Sciences and Sport, University of Stirling, Stirling, FK9 4LA, UK; Physical Activity for Health Group, Department of Psychological Sciences and Health, University of Strathclyde, Glasgow, G1 1QE, UK; MRC/CSO Social and Public Health Sciences Unit, School of Health and Wellbeing, University of Glasgow, Glasgow, G12 8TB, UK; Physical Activity for Health Group, Department of Psychological Sciences and Health, University of Strathclyde, Glasgow, G1 1QE, UK; School of Pharmacy and Life Sciences, Robert Gordon University, Aberdeen, AB10 7GJ, UK; Institute of Applied Health Sciences, School of Medicine, Medical Sciences and Nutrition, University of Aberdeen, Aberdeen, AB25 2ZD, UK; Strathclyde Institute of Education, University of Strathclyde, Glasgow, G4 0LT, UK

**Keywords:** food and nutrition, obesity, physical activity

## Abstract

**Background:**

Policymaking regarding physical activity (PA) and diet plays an important role in childhood health promotion. This study provides a detailed examination of Scottish government and policy for child and adolescent PA and diet and discusses strengths and areas for improvement.

**Methods:**

Scottish policy documents (*n* = 18 [PA]; *n* = 10 [diet])—published in 2011–20—were reviewed for grading using an adapted version of the Health-Enhancing Physical Activity Policy Audit Tool Version 2.

**Results:**

There is clear evidence of leadership and commitment to improving PA and diet and tackling obesity in children and adolescents. The allocation of funds and resources for policy implementation has increased substantially over the past decade. Progress through early key stages of public policymaking—policy agenda and formation—has improved. However, there is limited information on later key stages, including policy monitoring and evaluation.

**Conclusions:**

Childhood PA and diet are a clear priority in Scotland, and PA and diet policies clearly support the desire to achieve other goals, including reducing inequalities and increasing active travel in Scotland. Nonetheless, future policies should be further strengthened through clear(er) plans of implementation, and monitoring and evaluation to support their societal impact.

## Introduction

Physical activity (PA) and a healthy diet play an important role in children and adolescents’ current and future health and wellbeing. Participation in regular moderate- to vigorous-intensity physical activity (MVPA) has cognitive, physical and mental health benefits for children and young people, including improved academic performance, improved physical fitness and fewer symptoms of depression.^1^ Similarly, good nutrition profoundly impacts children’s growth and development with long-term benefits for both individuals and societies. Therefore, it is crucial for governments to have policies that prioritize PA opportunities and support healthy diets. Policy actions, as outlined in the World Health Organization’s (WHO) Global Action Plan for the Prevention and Control of Noncommunicable Diseases^2^ and the Physical Activity Strategy for the WHO European Region^3^ can support the achievement of Sustainable Development Goals (SDGs).^4^ PA and diet are intrinsically linked to several SDGs, including ensuring healthy lives and promoting wellbeing for all, quality education, gender equality, reduced inequalities and sustainable cities and communities.^4^^,^^5^ In Scotland, the SDGs are contextualized and implemented through the National Performance Framework, which sets out to develop ‘a more successful country with opportunities for all of Scotland to flourish through increased wellbeing, and sustainable and inclusive economic growth’. Moreover, this framework comprises a set of interrelated national outcomes to which relevant SDGs are mapped. One of these national outcomes is health, which covers multiple indicators including PA and healthy weight. In 2020, the Scottish Government published a national review on current evidence regarding actions and progress towards meeting the SDGs, and the challenges and next steps.^6^ The review indicates a poor performance on both PA and diet indicators amongst children and youth, with a significant proportion of children not meeting the recommendation of an average of 60 min of MVPA across the week, nor the recommended minimum five portions of fruit and vegetables daily.^7^^,^^8^

As part of the Global Matrix initiative launched by the Active Healthy Kids Global Alliance (AHKGA), the 2021 Active Healthy Kids Scotland Report Card has summarized the status of PA and health indicators in Scottish children and adolescents prior to the COVID-19 pandemic.^9^ These indicators include overall PA, sedentary behaviour (SB), diet, and government and policy, amongst others. The focus on government and policy in the context of PA and diet in children and adolescents is important considering the increasing international evidence describing persistently low PA and high SB levels, poor diet and high prevalence of childhood obesity. The need for evaluating the effectiveness of policies that aim and/or promise to address these issues is increasing. Therefore, this study aims to provide a comprehensive evaluation of evidence informing Scottish government and policies addressing PA and diet, and highlight strengths and areas for improvement in existing Scottish policies.

## Methods

The AHKGA defines the Government and Policy indicator as ‘any governmental body with authority to influence physical activity opportunities or participation of children and youth through policy, legislation or regulation’. As such, the Scottish Government website (https://www.gov.scot/) was searched for existing policy documents for both PA and diet. In addition to this, Google and Google Scholar searches were conducted using renditions of the following search phrases for PA: ‘physical activity policies Scotland’, ‘physical education policy Scotland’ and ‘Scotland physical activity’, and for Diet: ‘diet policy Scotland’, ‘dietary policy Scotland’, ‘food policy Scotland’ and ‘obesity policy Scotland’. Lastly, the Report Card team met regularly to discuss any other policy documents that would be worth considering. Policy documents were considered for grading if the policy was relevant for the ‘catchment’ time of the 2021 Report Card. This included policies published after the release of the 2018 Report Card,^10^ as well as policies published prior to 2017 but with an update within this period, or policies that were deemed current. To be consistent with previously published Report Cards, grades are based on data pertaining to children and young people from birth to 18 years. In the context of the Government and Policy grade, policy documents and instruments for the same age band were considered. Importantly, policy documents and initiatives published in response to the COVID-19 pandemic were not considered for grading.

Policy documents that contained both PA and diet elements were considered separately for their PA and diet components. It is relevant to note that one document considered for the PA component, the UK Chief Medical Officer (CMO) Guidelines for Physical Activity,^7^ is not housed within the Scottish Government: Scotland is one of four nations that make up the UK, and while health is devolved to Scotland, the UK CMO Guidelines for PA is an example of a collaboration between devolved Governments, making the guidelines pertinent to Scotland and relevant to include.

Unlike previously published report cards, where no tool was used to score data for the Government and Policy indicator, the Health-Enhancing Physical Activity (HEPA) Policy Audit Tool Version 2 (PAT v2^11^), as adapted, and described by Ward and colleagues^12^ for the analysis of policies within the 2018 Active Healthy Kids Wales Report Card,^13^ was used. This tool is useful in assessing and collating information for a variety of relevant policy characteristics (shown in Table 1). In addition, this tool allows users to not only descriptively appraise policies, but also provide a quantitative metric (as a score out of 100, where an ‘A’ means that *We are succeeding with a large majority of children, 87–93%*; an ‘F’ means that *We are succeeding with very few children, <20%*; see Supplementary Table for the full description). In addition to the score provided, and in accordance with the AHKGA, the policy documents and instruments were also considered according to the following three benchmarks: (i) evidence of leadership and commitment in providing PA/healthy diet opportunities for all children and youth; (ii) allocated funds and resources for the implementation of PA/healthy diet promotion strategies and initiatives for all children and youth; (iii) demonstrated progress through the key stages of public policymaking (i.e. policy agenda, policy formation, policy implementation, policy evaluation and decisions about the future). The policy evaluation and grade (within the Report Card) was shared with stakeholders and feedback on the Report Card was invited prior to publication.^9^

**Table 1:** Scoring rubric adapted from the HEPA Policy Audit Tool Version 2 (HEPA PAT v2) with scores for the Government & Policy Indicator (PA and diet policy) from the 2021 Active Healthy Kids Scotland Report Card

Criterion					Score	
					PA	Diet
**Number and breadth of relevant policies** Policies/strategies/action plans that reference physical activity	***Policy number***	***Score***	***Policy breadth (no. of sectors)***	***Score***	9 (4 + 5)	6 (3 + 3)
	*20+*	*5*	*10+*	*5*		
	*15–19*	*4*	*8–9*	*4*		
	*10–14*	*3*	*6–7*	*3*		
	*5–9*	*2*	*4–5*	*2*		
	*1–4*	*1*	*1–3*	*1*		
	*0*	*0*	*0*	*0*		
**Identified supporting actions** Strategic documents with specific actions that promote physical activity	***No. of policies with identifiable actions***		***Score***		12	8
	*Actual number of documents to maximum of 20*		*0–20*			
**Identified accountable organization(s)** Discreet organizations specifically identified as responsible for delivery of actions	***Proportion (%) of policies with identified responsibilities for delivery of actions***		***Score***		15	25
	*100%*		*25*			
	*80%*		*20*			
	*60%*		*15*			
	*40%*		*10*			
	*20%*		*5*			
	*0*		*0*			
**Identifiable reporting structures** Strategic documents with explicit reporting systems including frequency and format of reports	***Proportion (%) of policies with identified systems for reporting delivery of actions***		***Score***		5	5
	*100%*		*15*			
	*75%*		*12.5*			
	*67%*		*10*			
	*50%*		*7.5*			
	*33%*		*5*			
	*20%*		*3*			
	*0*		*0*			
**Identified funding** Explicit references to funding to support identified actions	***Proportion (%) of policies with identified funding sources***		***Score***		2	10
	*100%*		*20*			
	*75%*		*15*			
	*50%*		*10*			
	*25%*		*5*			
	*10%*		*2*			
	*0*		*0*			
**Monitoring and evaluation plan** Explicit reference to monitoring and evaluation of progress and impact of the policy	***Proportion (%) of included policies with identified systems for monitoring and evaluation***		***Score***		2.5	5
	*100%*		*10*			
	*75%*		*7.5*			
	*50%*		*5*			
	*25%*		*2.5*			
	*10%*		*1*			
	*0*		*0*			
**Total score**					**45.5**	**59**

No ethical approval was sought for this study, as the study relied exclusively on publicly available sources.

## Results

A narrative summary and explanation for the provided score for each criterion of the HEPA PAT v2 follows. Table 1 provides a breakdown of how each score was allocated and Table 2 provides an overview of the policy documents considered.

**Table 2:** Policy documents considered for the Government Indicator for the 2021 Active Healthy Kids Scotland Report Card

Name/title of policy document	Publication year(s)	Identified supporting actions	Identified accountable organization(s)	Identifiable reporting structures	Identified funding	Monitoring and evaluation plan
Obesity Route Map—Action Plan	2011; 2020	**✓**	**✓**	**✓**	**—**	**✓**
National parenting strategy	2012	**✓**	**✓**	**✓**	**✓**	**—**
Play Strategy for Scotland: Our Action Plan	2013	**✓**	**✓**	**—**	**—**	**—**
Let’s get Scotland Walking: The National Walking Strategy, Action Plan 2016–2026	2014; 2019	**✓**	**✓**	**✓**	**—**	**✓**
Cycling Action Plan for Scotland: 2017–2020	2017	**✓**	**✓**	**✓**	**✓**	**✓**
Public Health Priorities for Scotland	2018	**✓**	**✓**	**—**	**—**	**—**
A healthier future: Scotland’s diet and healthy weight delivery plan	2018	**—**	**—**	**—**	**—**	**—**
A More Active Scotland: Scotland’s Physical Activity Delivery Plan	2018	**✓**	**✓**	**✓**	**—**	**✓**
Restricted Roads (20 mph Speed Limit)—(Scotland) Bill: Policy memorandum	2018	**✓**	**✓**	**✓**	**—**	**—**
Sport for Life: A Vision for Scotland	2019	**✓**	**—**	**✓**	**—**	**✓**
Included, engaged, and involved. Part 1: promoting and managing school attendance	2019	**—**	**—**	**—**	**—**	**—**
UK Chief Medical Officers’ Physical Activity Guidelines	2019	**✓**	**—**	**—**	**—**	**✓**
Protecting Scotland’s Future: The Government’s Programme for Scotland 2019–20	2019; 2020	**—**	**✓**	**—**	**✓**	**—**
Realizing the ambition: Being Me: National practice guidance for early years in Scotland	2020	**—**	**✓**	**—**	**—**	**—**
Recommendation report from the Scottish Government’s Body Image Advisory Group on Good Body Image	2020	**✓**	**✓**	**—**	**—**	**—**
Scotland and the Sustainable Development Goals: A national review to drive action	2020	**—**	**—**	**—**	**—**	**—**
Update to the Climate Change Plan: 2018–2032	2020	**—**	**✓**	**—**	**—**	**—**
Scotland’s National Outdoor Play & Learning Position Statement	2020	**✓**	**—**	**—**	**—**	**—**
Better eating, better learning: a new context for school food	2014	**✓**	**✓**	**—**	**—**	**—**
Beyond the School Gate—Improving Food Choices in the School Community	2014	**✓**	**✓**	**—**	**✓**	**—**
Recipe for Success: Scotland’s national food and drink policy, becoming a Good Food Nation	2014	**✓**	**✓**	**—**	**✓**	**—**
Universal Free School Meals	2015	**✓**	**✓**	**✓**	**✓**	**✓**
Criteria for healthcare retail standard	2015	**✓**	**✓**	**✓**	**—**	**✓**
Revised Dietary Goals for Scotland	2016	**—**	**✓**	**—**	**—**	**✓**
A fairer, healthier Scotland: A strategic Framework for Action (2017–2022)	2017	**—**	**✓**	**✓**	**—**	**✓**
Fair Food Fund, under Poverty and Social Justice Policy (2017)	2017	**✓**	**✓**	**—**	**✓**	**—**
Soft Drinks Industry Levy (2018), under The Finance Act (2017)	2018; 2017	**✓**	**✓**	**—**	**—**	**—**
A healthier future: Scotland’s diet and healthy weight delivery plan (Superseded Obesity Route Map (2010))	2018	**✓**	**✓**	**—**	**✓**	**—**

### Number and breadth of relevant policies

There were 18 national policies/strategies/action plans/guidelines that mentioned and/or addressed PA and 10 related to diet in children. These policy documents represented a variety of directorates (‘sectors’) and departments within the Scottish Government (14 for PA, 6 for diet, out of 43). Scores of 9 and 6 (out of 10) were provided for PA and diet, respectively.

### Identified supporting actions

Twelve of the 18 PA policy documents mentioned specific actions to promote PA. In some cases, the actions are for the purpose of improving/increasing PA; for others, PA is promoted to meet another goal (e.g. reduce overweight/obesity, active travel). Eight of the 10 diet policy documents mentioned specific actions to improve diet or tackle obesity. Scores of 12 and 8 (out of 20) were provided for PA and diet, respectively.

### Identified accountable organization(s)

Twelve of the 18 PA policy documents (67%) mentioned partner organizations. These partners included Paths for All, sportscotland, Cycling Scotland, Sustrans, Actify and The Daily Mile. A score of 15 was allocated. All 10 of the diet policy documents (100%) had identifiable accountable organizations, including Local Authorities, the NHS, Food Standards Scotland, and Food and Drink industry bodies. The maximum score of 25 was allocated.

### Identifiable reporting structures

Seven of the 18 PA policy documents (39%) identified monitoring and reporting systems, although the structure of this information was highly variable between policy documents. A score of 5 was allocated. Only 3 of the 10 diet policies (30%) identify monitoring and reporting systems. A score of 5 was allocated.

### Identified funding

Three of the 18 PA policy documents (17%) mentioned or reported funding that is specifically allocated to promoting/increasing PA. A score of 2 was allocated. Five out of 10 diet policies (50%) had identified their funding. Many of the sources listed were vague. A score of 10 was allocated.

### Monitoring and evaluation plan

Six of the 18 PA policy documents (33%) explicitly referenced their plan for monitoring and evaluation. It is promising to note that there were some policies that had been updated or had progress reports published (Table 2). A score of 2.5 was allocated. Four of the 10 diet policies (40%) had a published monitoring and evaluation plan. Many policies have promised evaluation plans, but they have not yet been published. A score of 5 was allocated.

Overall, the government indicator for PA was graded ‘C−’, which corresponds with a total score of 45.5/100 or 45.5%. The indicator for diet was graded ‘C+’ overall, corresponding to a score of 59/100 or 59% (see Supplementary Table S1).

## Discussion

### Main finding of this study

This study aimed to evaluate Scottish policies for PA and diet in children and adolescents using a formal and systematic method. The findings indicate that Scotland has many creditable policies that lay a firm foundation for the improvement, promotion, and support of PA and healthy diets in children. The grades awarded (based on the AHKGA methodology used for the 2021 Active Healthy Report Card for the Government and Policy indicator) were ‘C−’ and ‘C+’ for PA and diet, respectively.

### What is already known on this topic

Policies tend to include limited information on implementation, and monitoring and evaluation stages. Importantly, this is noted in previous policy evaluations carried out in previous Active Healthy Kids Scotland report cards in 2013,^14^ 2016^15^ and 2018,^10^ where the evaluation (as well as the grade awarded) over the past decade has been broadly similar. It should be noted that this is the first time that separate grades have been provided for PA and diet policy. Over this period, there have been multiple policies targeting PA and/or diet, but there are persistent concerns and identifiable gaps when it comes to implementation and evaluation and funding. In the present policy evaluation, only 3 out of 18 PA and only 5 out of 10 diet policies mentioned funding allocated. These gaps highlight important areas that need to be addressed in policy documents.

### What this study adds

The present study found instances where policy lacks evidence of evaluation of either process or outcome, e.g. key policy targets in relation to time spent in MVPA in children and adolescents are undermined by the fact that the main surveillance tool, the Scottish Health Survey,^16^ does not measure MVPA,^9^ and detailed up-to-date dietary information for children in Scotland is lacking. This is important because it is difficult to determine the impact of a policy if evidence of monitoring and evaluation is lacking. Other concerns over the past decade include the evidence that PA policies do not address all children and adolescents (as specified in the AHKGA policy benchmark), but focused on adolescents and girls, while often overlooking other specific groups.^17^^,^^18^ Future policy documents would benefit from giving specific attention to other vulnerable or underrepresented groups who are likely to benefit even more from robust PA and diet policies (e.g. children with chronic conditions, children with disabilities, children from different ethnic groups, children with specific dietary requirements). Furthermore, future policy documents would benefit from making explicit reference(s) to monitoring and evaluation of progress to determine impact of the policy. For instance, the *Cycling Action Plan for Scotland: 2017–2020* is one of two policy documents to ‘tick all the boxes’ (see Table 2) and one of ten explicitly referencing monitoring and evaluation. This *Cycling Action Plan* has, arguably, influenced a number of more recent developments and successes (which will be reviewed in future publications of the Scottish Active Healthy Kids Report Card), including but not limited to the implementation of the *Free Bikes Pilots for School Age Children Who Cannot Afford Them* initiative,^19^ and an upward trend between 2013 and 2022 for children cycling to school, according to the most recently available *Hands Up Scotland Survey.*^20^ The other high-scoring policy of *Universal Free School Meals (2015)* has been monitored and evaluated (focusing on processes) since its implementation,^21^^,^^22^ providing support for further roll-out beyond the initial primary 1 to 3 stage, with free school meals now provided up to primary 5. There has been a general decline in uptake of school meals; however, there is a higher uptake of free school meals amongst primary 1 to 5 pupils who receive universal free school meals versus means-tested primary 6 to 7 pupils (72% versus 42% in 2023).^23^ The policy has been criticized for lack of impact evaluation, although there is evidence from other countries that universal free school meal provision increases uptake in those who are already eligible, due to reduced stigma and that providing free school meals to vulnerable children can improve health and learning outcomes.^24–26^

For children aged ≤5 years, the policies and position statements addressing play are particularly welcome, given that active and outdoor play is positively associated with PA.^27^^,^^28^ There are comprehensive policies that were not evaluated in this study (due to having no explicit mention of PA) but may enable active and outdoor play. For example, in Scotland, the expansion of free childcare hours (from 16 to 30 h per week) for all 3- and 4-year-olds and eligible 2-year-olds, as well as a focus on increasing outdoor nature-based provision, could support more children across the socio-economic spectrum to engage in active and outdoor play, as well as through providing healthy and balanced nutritious meals and snacks.^29^^,^^30^ Similarly, the proposal to extend early learning and childcare funding to parents who choose to defer their child’s P1 start (the first year of formal schooling in Scotland) from August 2023 may also influence PA and diet. This policy affords parents and caregivers across the socio-economic spectrum more freedom to choose their child’s education path with less/no financial ‘penalty’, as state schooling in Scotland is fully subsidized by the government. This is relevant because it may enable children to stay in a play-promoting environment for longer in comparison to primary school, where play opportunities may be less frequent. Finally, the additional financial support for parents of children under 5 years through the Best Start Grant could potentially enable parents to provide suitable clothing for poor weather, which is an important barrier to enable children to play and engage in PA, while also supporting low-income families to buy healthy foods.^31^ Having comprehensive and interconnected policies that target multiple levels of the system could not only benefit the PA levels and diets of young children presently, but also impact older children and the wider population.

### Limitations of this study

The present study aimed to provide a more comprehensive and objective evaluation of PA and diet policies for children and adolescents in Scotland than in previous years by using a policy audit tool. The HEPA PAT v2 is intended to provide a more systematic and comprehensive appraisal of policy. The experience of using it in the present Report Card is worth discussing, as the grades produced by the two methods (i.e. expert consensus used in previous report cards versus the HEPA PAT v2) are similar. The HEPA PAT v2 has currently been used by few research groups to evaluate children’s PA policy within the context of the AHKGA (e.g. Wales^13^). To the best of our knowledge, it has not previously been applied to diet policies. Although the HEPA PAT v2 is a useful and informative tool for policy evaluation, its use in the Scottish Report Card 2021 has identified some potential modifications and considerations, which may improve its use in the future (for Report Cards or other purposes). Firstly, the HEPA PAT v2 focuses on the assessment of policy that exists, thus, policy that does not yet exist cannot be evaluated. For instance, the absence of Scottish policies in important areas for child and adolescent public health, such as time spent in sedentary behaviour and/or sleep, is a concern, especially considering the evidence reporting the detrimental effects of excessive screen time and poor sleep quality/quantity on adolescents^32^ and the publication of the WHO guidelines on time spent in these behaviours in children.^33^^,^^34^ Notably, the WHO guidelines on SB, screen time and sleep (specifically for children <5 years) were not accepted by the CMOs in the UK.^35^ This absence in policy was (or would be) identified and highlighted using the previous AHKGA methodology (i.e. expert consensus). The HEPA PAT v2 may also be viewed as a time-consuming ‘counting exercise’, given its methodology, and may lead to assumptions that policies that ‘tick all the boxes’ will automatically be more impactful than policies that tick fewer boxes. There are also notable issues with under-estimated scoring when using the HEPA PAT v2; e.g. for *Identified Funding*, if the *Proportion (%) of Policies with Identified Funding Sources* was 33%, this section was scored as 25% (see Table 1). Therefore, we would argue that there is value in approaching policy evaluation with pragmatism and combining HEPA PAT v2 and other methods, especially those that are repeatable, for the sake of consistency, objectivity and rigour. Lastly, the HEPA PAT v2 has a focus on targeted policy aspects and this does not lend itself to overarching policy objectives. For instance, Scottish public health policy has for many years—to its credit—focused on reducing inequalities in health. This is, in principle, not a specific PA or dietary policy objective and is therefore not easily incorporated into policy appraisal but is deserving of attention.

## Conclusion

This paper reports a systematic evaluation of Scotland’s many creditable policies targeting both PA and diet for children, and highlights the importance of policy evaluation for PA, diet and other facets of public health. The creditable policies reviewed in this paper are indicative of the prioritization of PA and healthy diets, not only for the health of Scottish children, but as a means for Scotland to achieve other goals (e.g. active travel supports climate change goals; good nutrition is intrinsically linked with achievement of the Sustainable Development Goals). In addition, there is evidence of good links between the policies and organizations accountable for implementation. Future policy documents pertaining to PA and diet, as well as public health more generally, would be strengthened by having clear(er) plans of implementation, monitoring, evaluation and impact.

## Supplementary Material

supp_table_scot_policy_paper

## Notes

**Simone A. Tomaz**, Lecturer

**John J. Reilly**, Professor

**Avril Johnstone**, Research Associate

**Adrienne Hughes**, Lecturer

**Jenni Robertson**, Research Assistant

**Leone C.A. Craig**, Lecturer

**Farid Bardid**, Lecturer
