# Supplementary material for: A systematic evaluation of physical activity and diet policies in Scotland: results from the 2021 Active Healthy Kids Report Card
Source: J Public Health (Oxf). 2024 Feb 22;46(2):e240–7. doi: 10.1093/pubmed/fdae022 (PMC11141590; doi:10.1093/pubmed/fdae022)
Supplement: supp_table_scot_policy_paper [file supp_table_scot_policy_paper.docx]

**Supplementary Table: Active Healthy Kids Global Alliance (AHKGA) grading scheme**

| **Grade** | **Percentage** | **Explanation (where applicable)** |
| --- | --- | --- |
| A+ | 94-100% |  |
| A | 87-93% | We are succeeding with a large majority of children |
| A- | 80-86% |  |
| B+ | 74-79% |  |
| B | 67-73% | We are succeeding with well over half of children |
| B- | 60-66% |  |
| C+ | 54-59% |  |
| C | 47-53% | We are succeeding with about half of children |
| C- | 40-46% |  |
| D+ | 34-39% |  |
| D | 27-33% | We are succeeding with less than half of children |
| D- | 20-26% |  |
| F | <20% | We are succeeding with very few of children |
| INC | - | Incomplete Grade, where [Scottish] data were not available or were insufficient/inadequate to assign a grade |
